# The Role of p38 MAPK in the Aetiopathogenesis of Psoriasis and Psoriatic Arthritis

**DOI:** 10.1155/2013/569751

**Published:** 2013-09-12

**Authors:** Athanasios Mavropoulos, Eirini I. Rigopoulou, Christos Liaskos, Dimitrios P. Bogdanos, Lazaros I. Sakkas

**Affiliations:** ^1^Cellular Immunotherapy and Molecular Immunodiagnostics, Institute of Research and Technology Thessaly, 41222 Larissa, Greece; ^2^Division of Transplantation Immunology and Mucosal Biology, Institute of Liver Studies, King's College London School of Medicine at King's College Hospital, Denmark Hill Campus, London SE5 9RS, UK; ^3^Department of Medicine, Faculty of Medicine, School of Health Sciences, University of Thessaly, Biopolis, 41110 Larissa, Greece; ^4^Center of Molecular Medicine, Old Dominion University, 23529 Monarch Way, Norfolk, VA, USA; ^5^Department of Rheumatology, Faculty of Medicine School of Health Sciences, University of Thessaly, Biopolis, 41110 Larissa, Greece

## Abstract

The pathogenetic mechanisms responsible for the induction of immune-mediated disorders, such as psoriasis, remain not well characterized. Molecular signaling pathways are not well described in psoriasis, as well as psoriatic arthritis, which is seen in up to 40% of patients with psoriasis. Signaling pathway defects have long been hypothesized to participate in the pathology of psoriasis, yet their implication in the altered psoriatic gene expression still remains unclear. Emerging data suggest a potential pathogenic role for mitogen activated protein kinases p38 (p38 MAPK) extracellular signal-regulated kinase 1/2 (ERK1/2), and c-Jun N-terminal kinase (JNK) in the development of psoriasis. The data are still limited, though, for psoriatic arthritis. This review discusses the current data suggesting a crucial role for p38 MAPK in the pathogenesis of these disorders.

## 1. Introduction 

Psoriasis is a chronic inflammatory skin disease affecting 1-2% of the population. Clinically, skin lesions are characterized by erythematous plaques covered by scales and pathologically by keratinocyte hyperproliferation and altered differentiation, inflammatory infiltrates, and neovascularization [1–4]. Up to 40% of patients with psoriasis develop an inflammatory arthritis called psoriatic arthritis (PsA) [5, 6].

The mechanisms responsible for skin lesions in psoriasis and the development of PsA remain elusive [7]. Nevertheless, a wealth of data supports the notion that specialized components of the immune system play an important role in the pathogenesis of these disorders [8–10]. Thus, psoriatic patients have elevated levels of circulating neutrophils, and specialized macrophages and dendritic cells appear early in skin lesions followed by activated natural killer cells and T cells that sustain a loop with distinctive Th1- and Th17-mediated pathology [11–17] (Table 1). PsA is also characterized by pronounced T- and B-cell infiltrates, synovial hyperplasia, and angiogenesis in the synovial membrane, as well as by overexpression of inflammatory cytokines and proteases [18, 19].

Cytokines, such as tumor necrosis factor- (TNF)-*α* and interferon-*γ* (IFN-*γ*), interleukin- (IL)-1*β*, IL-6, and IL-8, and chemokines, such as CXCL9, CXCL10, CXCL11, and cyclooxygenase-2 (COX-2), are involved in the initiation and perpetuation of psoriatic lesions [33–36]. Th17 cells with their signature cytokine IL-17 are also involved in psoriasis and PsA [37, 38]. Recently, additional cytokines, namely, IL-21, IL-22, IL-23, and IL-27, as well as certain inflammatory biomarkers, have been implicated in psoriatic pathologies [39–45] (Table 1).

The use of cytokine antagonists is an important therapeutic advance in the current management of PsA and plaque psoriasis, and new studies evaluate the efficacy and safety of new cytokine antagonists in these disorders [46]. The novel IL-23/Th17 axis has attracted much attention with the successful application of ustekinumab, a monoclonal antibody against IL-12 and IL-23, in psoriasis and PsA [47–49]. Some success was also reported with anti-IL-17A and anti-IL-22 agents in animal models of psoriasis [50]. Inhibition of IFN-*γ* expression is of special interest, since IFN-*γ* has been recently shown to enhance IL-22 and IL-23 expressions and subsequently induce Th17 cells in psoriatic lesions. A single intradermal injection of IFN-*γ* can induce an inflammatory state in both nonlesional psoriatic and healthy skin [51–55]. 

## 2. The Role of p38 MAPK in Psoriasis

Signaling pathway defects have long been hypothesized to participate in the pathology of psoriasis, yet their implication in the altered psoriatic gene expression still remains elusive [56, 57]. The application of immunohistochemistry and Western blotting techniques has provided some information regarding the signaling cascades in psoriatic skin. These experiments identified mitogen-activated protein kinases p38 (p38 MAPKs), extracellular signal-regulated kinase 1/2 (ERK1/2), and c-Jun N-terminal kinase (JNK) to be involved in the pathogenesis of psoriasis [20–22, 58, 59] (Table 2). The MAPK kinases constitute an important set of three signaling pathways, namely, p38, ERK1/2, and JNK, which control several important functions within the cell, such as cell proliferation, differentiation, gene expression, and apoptosis [60]. The extensive thickening of the epidermis is indicative of imbalance in the homeostasis between proliferation and apoptosis. Indeed, the expression of various apoptosis-related molecules is increased in the psoriatic hyperproliferative epidermis [61].

Kinase assays further confirmed the increased activation of p38 and demonstrated increased activity of the p38 isoforms p38*α*, p38*β*, and p38*δ* in lesional compared to nonlesional psoriatic skin [20]. Phosphorylated p38 was widely detected in lesional psoriatic epidermis and exhibited a distinct nuclear localization indicative of the kinase participation in the induction of active gene expression. Recently, the antimicrobial peptide S100A8, known to be upregulated in lesional psoriatic skin, was found to be regulated by a p38-MAPK-dependent mechanism [29]. Similarly, p38-dependent expression was demonstrated for the antimicrobial peptides cathelicidin, human *β*-defensin-2, human *β*-defensin-3, and S100A7 in human keratinocytes [30].

The p38 MAPK signaling pathway is a critical mediator in the regulation of both cellular and humoral autoimmune responses [62]. Usually initiated by cellular stresses, T-cell receptor or inflammatory cytokines, p38 MAPK regulates cytokine gene expression by means of transcriptional and posttranscriptional mechanisms, such as stabilization of mRNA transcripts [63–65]. Defects in p38 MAPK pathway can explain the increased expression of proinflammatory cytokines in several immune-mediated diseases, and several pharmaceutical companies have invested heavily in the development of agents that inhibit p38 MAPK activation [66–68]. An increasing number of novel p38 MAPK inhibitors have been tested in experimental models and clinical trials and have advanced our knowledge on the role of p38 MAPK [69].

Therapeutic inhibition of p38 MAPK pathway is mainly based on the notion that natural-negative-feedback mechanisms exist to guarantee that MAPKs are not activated ad infinitum. In this regard, MAP kinases can themselves induce different types of protein phosphatases called dual-specificity phosphatases (DUSPs). DUSPs dephosphorylate MAP kinases and cease their function [70, 71]. Interestingly, the p38 MAPK-negative-feedback mechanism provided by DUSP1 seems to be impaired in psoriasis since DUSP1 mRNA expression was significantly downregulated in psoriatic skin lesions compared to nonlesional psoriatic skin [26].

MAPKs are activated by phosphorylation of both threonine and tyrosine residues, and in turn they phosphorylate other downstream intracellular kinases and transcription factors. One of the downstream targets of p38 MAPK signaling cascade is MAPK-activated protein kinase 2 (MK2). Increased levels of activated MK2 were found in psoriatic lesions [72]. The activity of MK2 was located in the psoriatic epidermis but not in nonlesional psoriatic skin. Additionally, keratinocytes transfected with MK2-specific small interfering RNA had a significant decrease in the MK2 expression, and subsequently a significant reduction in the protein expression of IFN-*γ*, TNF-*α*, IL-6, and IL-8. The mechanism by which p38 MAPK mediates its regulatory effects through downstream kinases has been studied in cells isolated from mice with deleted MK2 [73]. Particularly interesting characteristics of MK2 knockouts are their increased survival and increased stress resistance upon LPS challenge. These mice are deficient in the LPS-induced biosynthesis of several proinflammatory cytokines regulated by p38, including TNF-*α*, IFN-*γ*, IL-6, and IL-1. They survive LPS-induced endotoxic shock due to a reduction of almost 90% in the secretion of TNF-*α* [74]. MK2 has been regarded as a key molecule participating in host defense against intracellular bacteria through regulation of both TNF-*α* and IFN-*γ* production [75, 76]. 

Mitogen- and stress-activated protein kinase 1 (MSK1) is another downstream target of both p38 and ERK1/2 MAPKs which regulates the expression of pro-inflammatory cytokine genes through activation of transcription factors. Western blotting analysis revealed a consistent and significant increase in phosphorylated MSK1 (Ser376) in lesional psoriatic skin [24, 77]. Cultured human keratinocytes incubated with anisomycin or IL-1beta resulted in the phosphorylation of both p38 MAPK and MSK1 (Ser376), whereas MSK1 (Ser376) phosphorylation was inhibited by preincubation with p38 inhibitors or dimethyl fumarate [78]. In addition, transcription factors, such as cAMP/calcium responsive element binding protein (CREB) associated with cellular proliferation gene expression, are also phosphorylated in psoriatic skin [21]. Activation of CREB through ERK1/2 is directly linked with the expression of TNF-*α*, IL-6, and IL-8 [79]. These cytokines are also under direct regulation by the p38 pathway as well [80, 81]. p38 MAPK-induced phosphorylation of STAT-3 and of STAT-1 at serine 727 has also been demonstrated in lesional psoriatic skin [27, 28]. Thus, keratinocytes in the psoriatic epidermis are characterized not only by abnormal proliferation and apoptosis but also by increased expression of inflammatory cytokines [81]. This seems to be regulated by the same signals arising from the activation of MAPK signaling cascades of p38 and ERK1/2 [20, 23, 82].

## 3. The Role of p38 MAPK in PsA

Information about the involvement of MAPK signaling in the pathogenesis of PsA is very scarce despite the fact that activation of MAPKs, specifically p38 and downstream MK2, has been described in rheumatoid arthritis (RA) synovium and the collagen-induced arthritis model of RA [67, 74]. Inflammatory cytokines upregulated in psoriasis appear to be involved in the pathogenesis of PsA and other spondyloarthritides as well [41, 83, 84]. For example, the administration of infliximab, an anti-TNF-*α* agent, to patients with PsA improved patients with active PsA and persistently high serum TNF-*α* levels [85]. A significant reduction in several proinflammatory and modulatory cytokines has also been noted in psoriatic patients with or without arthritis after treatment with etanercept, another TNF-*α* inhibitor [86, 87]. Back in 2000, Danning et al. have linked elevated proinflammatory cytokines with NF*κ*B activation in PsA synovium [88]. More recent findings underlined the participation of both MAPK signaling and NF*κ*B activation in PsA synovium before and after treatment with etanercept [31] (Table 2). Activated p38 was present in both lining and sublining area of the synovial membrane, and p38 positive cells were detected in inflammatory infiltrates and perivascular zones. Activated ERK was mainly present in the sublining area and mononuclear cell infiltrates, whereas activation of JNK was observed in cells of the lining layer of the synovial membrane [31]. This is of particular interest since epidermal deletion of c-Jun and jun-B proteins also triggers psoriasis and psoriatic arthritis in mice [89]. In addition, IL-36*α* is upregulated in PsA and RA synovia and leads to IL-6 and IL-8 production by synovial fibroblasts through p38/NF*κ*B activation [32].

The synovial membrane of PsA is characterized by T-cell and B-cell infiltrates, synovial hyperplasia, angiogenesis, and overexpression of inflammatory cytokines. A better understanding of these cellular populations their signaling pathways, and associated gene expression is necessary in order to advance our knowledge on the pathogenesis of PsA and to successfully identify novel molecular therapeutic targets. Peripheral blood mononuclear cell (PBMC) analyses have also aided in providing gene expression profiles of patients with systemic autoimmune disorders, such as RA, multiple sclerosis, and systemic lupus erythematosus. One of such studies in PBMC of patients with PsA identified a unique gene expression signature with MAPK signaling members being among the genes with reduced levels of expression [90]. One interpretation could be that the reduced mRNA levels of certain MAPK pathway members create an imbalance that favors the development of proinflammatory cells in PsA. However, it should be noted that the activation status of MAPK pathway is regulated at the posttranslational level. There was also reduced expression of B-cell specific genes including those of cell activation and T-cell activation genes. These observations from peripheral blood need to be considered with caution in relation to infiltrating lymphocytes from psoriatic skin biopsies [91, 92]. Studies of skin biopsies have identified several upregulated proinflammatory genes including IL-1, IL-6, and IL-8. 

Yet, peripheral blood signature studies are of great importance and can provide a lot of information regarding new immune regulatory molecules [93]. For instance, we have optimized a PB flow cytometry-based assay that details cellular phenotypic status, signaling status, and gene expression analysis, all combined [94]. Applications of optimized protocols based on sensitive phospho flow cytometry have been recognised as promising alternatives for the investigation of the phosphorylation of p38 MAPK within different PBMC populations [95, 96]. Flow cytometry has been so far useful in revealing the phenotype of psoriatic lesion infiltrating cells and their secreted cytokines [11, 97]. However, there has been no information on the kinases and signaling pathways activated in the rare NK and NKT cells of PsA patients. We have previously shown that p38 MAPK regulates posttranscriptional IFN-*γ* gene expression in human NK and NKT cells [63]. This possibly occurs via an MKK6/p38/MK2-dependent mechanism for the stabilization of IFN-*γ* mRNA in NK and NKT cells and may play an important role in host defense as well. We have optimized the methodology for the successful application of phospho-specific flow cytometry in order to detect phosphorylated p38 MAPK within innate immune cells, such as NK and NKT [94, 98]. Because of the critical and bidirectional role of IFN-*γ* in inflammation and autoimmunity, the elucidation of the molecular mechanisms controlling its expression is the focus of ongoing research by our team [62]. 

As mentioned previously, NK and NKT cells appear as key players in the pathogenesis of psoriasis [12, 99, 100]. In fact, these cells play a significant role in regulating a number of autoimmune skin disorders [101, 102]. Decreased numbers of CD56+ cells have been detected in PB of patients with psoriasis, and impaired function has been documented in other autoimmune diseases, such as primary biliary cirrhosis, multiple sclerosis, systemic lupus erythematosus, RA, and type I diabetes [103–107]. Alterations in killer inhibitory receptors (KIRs) repertoire expression on NK cells and on T cells have also been associated with several autoimmune diseases including multiple sclerosis, type I diabetes, and psoriasis [108–110]. There is a significant depletion of PB NK and NKT numbers in psoriatic patients that is probably due to an increased accumulation of activated NK and T cells bearing NK-associated receptors in psoriatic lesions [103]. The assessment of MAPK expression levels in cell subpopulations from psoriatic patients would be very informative.

 Lesional T cells analyzed by flow cytometry express certain classical NK phenotypic markers, such as CD56, CD16, and CD94, and there is a significant positive correlation between circulating CD8+ CD94/NKG2A+ T cells and the severity index of the psoriatic skin lesions [11, 111]. Patterns of CD1d expression are also observed in keratinocytes *in vitro* and in human skin with psoriasis *in vivo *[99]. NKT cells can become activated in a CD1d-restricted fashion with subsequent proliferation and cytokine production, including IFN-*γ* and IL-4. The ability of CD1d-positive keratinocytes to activate NKT cells to produce IFN-*γ* could represent a mechanism that contributes to the pathogenesis of PsA, psoriasis, and other autoimmune skin disorders.

In conclusion, current data suggest that p38 MAPK plays a role in the pathogenesis of psoriasis. However, more studies are needed to further advance this interesting topic of research. At present, the information regarding p38 MAPK involvement in the pathogenesis of PsA is limited and by no means conclusive.

## Figures and Tables

**Table 1:** Innate and adaptive immunity mediators likely to be involved in psoriasis and psoriatic arthritis.

Psoriasis	Psoriatic arthritis
(i) Prominent lymphocytic infiltrate present in skin	(i) Prominent lymphocytic infiltrate present in joints

(i) TH1, TH17, TH22, NK, NKT, B cells (ii) Dendritic cells, macrophages	(i) TH1, TH17, TH22, NK?, NKT?, B cells (ii) Dendritic cells, macrophages?

(i) IL-12, IL-23, TNF-A, IFN-*γ*	(i) IL-12, IL-23, TNF-A, IFN-γ

(i) IL-6, IL-17A, IL-17F, IL-21, IL-22, IL-26, IL-27 (ii) Cathepsin K, psoriasin (iii) Keratin 17 (iv) Plasma YKL-40? (v) Purinergic receptor P2X7 (vi) Trem 1	(i) IL-6, IL-17A, IL-17F, IL-21, IL-22, IL-26, IL-27 (ii) Cathepsin K?, psoriasin? (iii) Keratin 17? (iv) Plasma YKL-40 (v) Purinergic receptor P2X7? (vi) Trem 1?

(i) MAP kinases ERK1/2, JNK, P38 (ii) MK2, DUSP-1 MSK-1, GSK-3*β* (iii) NFKB, JAK/STAT-3, CREB	(i) MAP kinases ERK1/2, JNK, P38? (ii) MK2?, DUSP-1?, MSK-1, GSK-3*β*? (iii) NFKB, JAK/STAT-3?, CREB

Question marks indicate inadequate data.

**Table 2:** Evidence for p38 MAPK involvement in psoriasis and psoriatic arthritis.

Diseases		References
Psoriasis	(1) p38 MAPK is phosphorylated in lesional psoriatic epidermis	[20–23]
	(2) Phosphorylated p38 is widely detectable in the keratinocyte nuclei indicative of the kinase strong participation in active gene expression	[21]
	(3) Among the p38 MAPK isoforms, p38alpha, p38beta, and p38delta are detectable in lesional psoriatic skin	[20]
	(4) p38-activated kinases MK-2 and MSK-1 are also phosphorylated in psoriatic lesional skin and regulate the production of proinflammatory cytokines such as TNF-*α*	[23–25]
	(5) Dual-specificity phosphatase 1 (DUSP1) is an important negative regulator of p38 MAPK activity DUSP1 mRNA expression is downregulated in psoriatic skin lesions compared with paired samples of nonlesional psoriatic skin	[26]
	(6) p38-MAPK induced Ser727 phosphorylation of STAT-1 and STAT-3 is detected in psoriatic skin	[27, 28]
	(7) p38-MAPK-dependent expression of cathelicidin antimicrobial peptide, human *β*-defensin-2, human *β*-defensin-3, S100A7, and S100A8	[29, 30]

Psoriatic arthritis	(1) Phosphorylated p38 MAPK is detectable in both lining and sublining synovial area	[31]
	(2) P38 positive cells are also detected in inflammatory infiltrates, in perivascular zones, and in the endothelium	[31]
	(3) IL-36*α* is upregulated in PsA and RA synovia and leads to IL-6 and IL-8 production by synovial fibroblasts through p38/NFkB activation	[32]

## Abbreviations

DUSP: Dual-specificity phosphatase
IL: Interleukin
MAPK: Mitogen-activated protein kinase
NK: Natural killer
NKT: NK T cells
PsA: Psoriatic arthritis
RA: Rheumatoid arthritis
TNF: Tumor necrosis factor.
